# Exon deletions and intragenic insertions are not rare in ataxia with oculomotor apraxia 2

**DOI:** 10.1186/1471-2350-10-87

**Published:** 2009-09-11

**Authors:** Veronica Bernard, Martina Minnerop, Katrin Bürk, Friedmar Kreuz, Gabriele Gillessen-Kaesbach, Christine Zühlke

**Affiliations:** 1 Institut für Humangenetik, Universität zu Lübeck, Lübeck, Germany; 2 Klinik für Neurologie, Universität Bonn, Bonn, Germany; 3 Institut für Neurowissenschaften und Medizin (INM-1), Forschungszentrum Jülich, Jülich, Germany; 4 Neurologische Klinik, Universität Marburg, Marburg, Germany; 5 Praxis für Humangenetik, Dresden, Germany

## Abstract

**Background:**

The autosomal recessively inherited ataxia with oculomotor apraxia 2 (AOA2) is a neurodegenerative disorder characterized by juvenile or adolescent age of onset, gait ataxia, cerebellar atrophy, axonal sensorimotor neuropathy, oculomotor apraxia, and elevated serum AFP levels. AOA2 is caused by mutations within the senataxin gene (*SETX*). The majority of known mutations are nonsense, missense, and splice site mutations, as well as small deletions and insertions.

**Methods:**

To detect mutations in patients showing a clinical phenotype consistent with AOA2, the coding region including splice sites of the *SETX *gene was sequenced and dosage analyses for all exons were performed on genomic DNA. The sequence of cDNA fragments of alternative transcripts isolated after RT-PCR was determined.

**Results:**

Sequence analyses of the *SETX *gene in four patients revealed a heterozygous nonsense mutation or a 4 bp deletion in three cases. In another patient, PCR amplification of exon 11 to 15 dropped out. Dosage analyses and breakpoint localisation yielded a 1.3 kb LINE1 insertion in exon 12 (patient P1) and a 6.1 kb deletion between intron 11 and intron 14 (patient P2) in addition to the heterozygous nonsense mutation R1606X. Patient P3 was compound heterozygous for a 4 bp deletion in exon 10 and a 20.7 kb deletion between intron 10 and 15. This deletion was present in a homozygous state in patient P4.

**Conclusion:**

Our findings indicate that gross mutations seem to be a frequent cause of AOA2 and reveal the importance of additional copy number analysis for routine diagnostics.

## Background

The autosomal recessive cerebellar ataxias (ARCA) represent a phenotypically and genetically heterogeneous group of neurodegenerative disorders mainly beginning before 20 years of age.

More than 20 different forms of ARCA have been described. Friedreich ataxia (FRDA) is the most frequent form in Europe followed by ataxia with oculomotor apraxia 2 (AOA2, OMIM #606002) [1]. AOA2 is genetically defined by mutations in the senataxin gene (*SETX*) (OMIM *608465) located on chromosome 9q34. Patients typically present with early onset ataxia (range: 3-30 years), peripheral axonal sensorimotor neuropathy with areflexia (> 90% of individuals), oculomotor apraxia (<50% of individuals), marked cerebellar atrophy on MRI, and slow progression [2]. Dystonic hand posture, choreic movements, and head or postural tremor are present in about 20% of individuals. Serum α-fetoprotein (AFP) concentration is elevated in >90% of affected individuals. In 50% of patients, serum cholesterol levels are increased. Serum creatine kinase (CK) may be abnormal in patients with severe amyotrophy.

Mutations in *SETX *were first reported in 2004 [2]. The *SETX *gene consisting of 26 exons (coding exons 3-26) encodes for senataxin, a 2677 amino acid protein containing a putative DNA/RNA helicase domain. This helicase domain possesses strong homology to yeast RNA helicase Sen1p. To date, at least 51 mutations within the *SETX *gene responsible for the AOA2 phenotype are known [2-14]. The majority are nonsense, missense, and splice site mutations as well as small deletions and insertions. AOA2 is allelic to ALS4, one form of amyotrophic lateral sclerosis with juvenile onset and autosomal dominant inheritance [6,15].

In addition to numerous mutations of single or few nucleotides, four cases with large gene rearrangements within *SETX *have been described in patients with AOA2. A ~20.6 kb deletion (intron 15 to intron 23) was identified in an Italian family [7], a ~10 kb duplication was found in a German patient [4] and in two families from Algeria a deletion of exon 7 and a deletion of exon 19 and 20 have been reported [14].

Samples of patients with gait instability, areflexia, neuropathy, cerebellar dysarthria, and oculomotor signs were screened for mutations in the *SETX *gene. In six patients, clinical diagnosis of AOA2 could be confirmed by sequence analyses [13]. Additionally, there was evidence for compound heterozygous deletions, insertions as well as homozygous deletions in AOA2 patients.

## Methods

### Patients

After obtaining informed consent, DNA and RNA were extracted from peripheral blood leukocytes using standard procedures. The study was approved by the Ethic Committee of the University to Lübeck (reference number: 09-041) in compliance with the Helsinki Declaration. Clinical data are summarized in Table 1.

**Table 1:** Clinical data at last presentation. MRI showed global cerebellar atrophy.

**patient**	**age**	**sex**	**DD**	**cerebellar ataxia**	**oculomotor apraxia**	**neuropathy**	**dystonia**	**pyramidal signs**	**dementia**	**MRI**	**AFP**	**cholesterol**	**CK**
**no.**	***[years]***		***[years]***						**(MMSE)**	**(atrophy)**	***[< 5 ng/ml]***	***[< 220 mg/dl]***	***[< 171 U/l]***

1	25	m	13	yes	yes	yes	no	no	No (27/30)	cerebellum	9.7	179	138

2	28	m	13	yes	yes	yes	no	no	No (30/30)	cerebellum	12.6	normal	193

3	29	m	17	yes	yes	yes	no	no	no	cerebellum	56	155	220

4	33	f	21	yes	no	yes	no	no	No (30/30)	cerebellum	32	n.a.	171

m/f = male/female

DD = disease duration

MMSE = Mini Mental State Examination (Folstein et al., J Psychiatr Res 1975; 1225.4:189-198)

AFP = alpha-fetoprotein

CK = creatin kinase

n.a. = not available

Patient P1 showed first signs of gait imbalance at 12 years of age. At the age of 25 years, he presented with marked ataxia and used a stroller. He also had oculomotor signs including oculomotor apraxia, cerebellar dysarthria, neuropathy with muscular atrophy and areflexia of upper and lower limbs. Serum AFP was elevated (9.7 ng/ml).

Patient P2 noticed first gait problems when he was 15 years old. At the age of 28, he presented with marked ataxia of gait and stance using a stroller for longer distances. He also had oculomotor apraxia, cerebellar dysarthria, neuropathy with muscular atrophy and areflexia of upper and lower limbs. Serum AFP was elevated (12.6 ng/ml).

In patient P3, ataxia started at the age of 12. Seventeen years later, he was not able to walk without support. Clinically, he showed neuropathy with amyotrophy including small hand muscles, pes cavus, and dysarthria. Serum AFP was clearly elevated (56 ng/ml).

Patient P4 experienced first gait disturbances at the age of 12. During the following years, she developed progressive atrophy of distal muscles in the lower limbs. At the age of 33, there was evidence of cerebellar ataxia and sensorimotor neuropathy. Muscular atrophy was generalized with involvement of hand and proximal hip muscles and a positive Trendelenburg's sign. Oculomotor testing revealed major fixation instability, downbeat and gaze evoked nystagmus, saccadic pursuit and bilateral sixth cranial nerve palsy. There was no oculomotor apraxia.

### Sequencing analysis

We screened for *SETX *mutations by direct sequencing of all 24 coding exons and flanking intronic sequences. PCR products were amplified using standard protocols (primer sequences available on request). After ExoSAP-IT treatment (USB Inc, Staufen, Germany) of the PCR products, sequencing reactions were performed using the BigDye Terminator v1.1 Sequencing Standard Kit (Applied Biosystems Inc, Darmstadt, Germany) and analysed on the automated capillary sequencer 3130 *xl *Genetic Analyser (Applied Biosystems Inc, Darmstadt, Germany).

### Haplotype analyses

In case of identical mutations in unrelated patients, haplotype analyses for the AOA2 region with the markers D9S159, D9S1831, D9S1863, D9S1847, D9S1830 and D9S1793 (NCBI database, primer sequences are listed in additional file 1) were performed. FAM-labelled PCR products were separated on automated capillary sequencer 3100-Avant Genetic Analyser (Applied Biosystems Inc, Darmstadt, Germany).

### Breakpoint localisation

Dosage analyses were performed on genomic DNA for the 24 coding exons of the *SETX *gene using the ABI 7300 Real Time PCR System (Applied Biosystems Inc, Darmstadt, Germany) in the presence of SYBR-Green (SYBR-Green I core reagent kit including AmpliTaq-GOLD polymerase, Applied Biosystems Inc, Darmstadt, Germany). The optimisation of the PCR reaction was performed according to the manufacturer's instructions (Applied Biosystems Inc, Darmstadt, Germany, User Bulletin 2 applied to the SYBR-Green I core reagent protocol) but scaled down to 25 μl per reaction.

Long-range PCR was performed using the Expand High Fidelity PCR System (Roche Diagnostics GmbH, Mannheim, Germany). Primers flanking the potential deletion were used. Whenever the amplification at genomic DNA level failed, the regions of possible deletion breakpoints were narrowed down by primer walking. Subsequently, PCR on genomic DNA across the deletion junctions were done using standard protocols (Primer sequences for breakpoint localisation are listed in additional file 2). PCR products have been verified by sequencing analysis.

### RNA analysis

For RNA analyses, total RNA was isolated from peripheral blood leukocytes using PAXgene kit according to the manual (PreAnalytiX, Hombrechtikon, Switzerland). Reverse transcriptase PCR was performed with the OneStep RT-PCR Kit (Qiagen, Hilden, Germany) according to manufacturer's recommendations. For RT-PCR, gene specific primers flanking the presumptive mutation were used (primers sequences are listed in additional file 3). Amplified products were separated on 0.8% agarose gels. Separated fragments were excised from the gel and eluted using the Perfectprep Gel Cleanup kit (Eppendorf, Hamburg, Germany). Extracted DNA was sequenced as mentioned above.

### Sequence Analysis

Sequence analyses were performed using the GenBank reference sequence (Accession number: NM_015046). Repetitive elements were analysed using RepeatMasker version open-3.2.7 .

## Results

### Detection of small mutations in the *SETX *gene by sequencing analysis

Sequencing the coding region of the *SETX *gene revealed heterozygous mutations in three patients (Table 2). In patient P4, PCR amplification of exon 11 to 15 failed.

**Table 2:** Mutations in *SETX*

**DNA**	**Alteration in DNA (Exon)**	**Alteration in Protein**	**Mutation Status**
Patient 1	c.4816C>T (10)	p.R1606X	compound heterozygous
	
	c.5401_5402ins1280bp	p.V1792_L1813del, p.V1792_M1850delinsV	

Patient 2	c.4816C>T (10)	p.R1606X	compound heterozygous
	
	c.5374+9369_5950-254del6107bp	p.V1792EfsX31, p.V1792_L2035del	

Patient 3	c.4633_4636delAGTG	p.S1545AfsX26	compound heterozygous
	
	c.5274+13396_6107-3547 del20729bp	p.V1759EfsX6	

Patient 4	c.5274+13396_6107-3547 del20729bp	p.V1759EfsX6	homozygous

Patient P1 and P2 were heterozygous for a C>T transition at position 4816 of the cDNA, that encodes a stop codon (c.4816C>T, p.R1606X). In both cases, heterozygosity for this nonsense mutation was also present in the maternal DNA. Haplotype analyses excluded a common founder for this nonsense mutation.

Patient P3 was heterozygous for a 4 bp deletion in exon 10 of the *SETX *gene (c.4633_4636delAGTG). This deletion leads to a frame shift and generates a premature stop codon (p.S1545AfsX26). Heterozygosity for this mutation was also confirmed in the mother.

### Searching for gross changes in the *SETX *gene by dosage analysis

The presence of heterozygous *SETX *mutations in three patients presenting with a clinical phenotype consistent with AOA2 suggested the implementation of dosage analyses to detect potential copy number variations. Reproducible aberrant signals were found in patients P1, P2, and P3 (data not shown). Patient P1 showed a 50% decrease for exon 12 compared to different exons of the gene. Patient P2 had reduced values for exon 12 to 14. In patient P3, a shifting to decreased gene dosage was detected for exon 11 to 15.

### Identification of mutations by long-range PCR and breakpoint analysis

**Figure 1:**
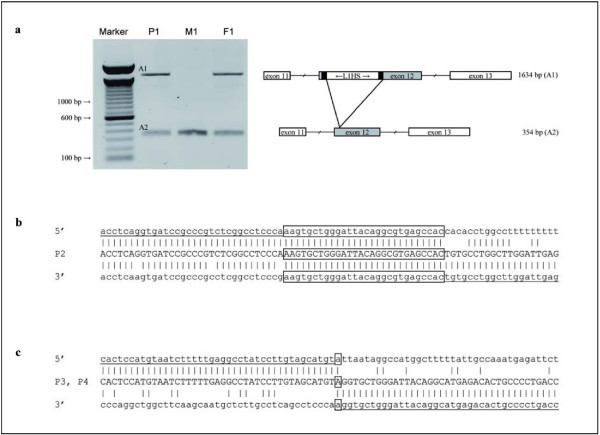
**Localisation of the Insertion and Deletion Breakpoints**. **(a) **Long range PCR products for patient P1, his mother M1, and his father F1 separated on a 0.8% agarose gel. Marker: 100 bp DNA Ladder. Amplicon A2 represents the wildtype fragment and A1 the PCR product with the L1HS insertion. Schematic drawing shows the L1HS insertion in exon 12. Exons are indicated as boxes, introns as interrupted lines. The L1HS insertion is flanked by a 15 bp target site duplication (black boxes). **(b) **Sequence of the breakpoint junction in patient P2 compared to control sequence. Homologous regions are boxed. **(c) **Sequence alignment of the breakpoint junction in patients P3 and P4 and the control 5' and 3' regions.

In patient P1, long-range PCR on genomic DNA using primers flanking exon 12 revealed the 354 bp wildtype fragment and an additional ~1.6 kb PCR product (P1, Figure 1a). The same pattern could be observed in the paternal DNA (F1), whereas the mother (M1) showed the wildtype fragment. Sequencing of the 1.6 kb PCR product indicated a 1.3 kb insertion within exon 12 (c.5401_5402ins1280bp). The insertion consists of a 5' truncated L1HS element. The first part of the 5' truncated L1HS element is orientated in antisense with respect to the disrupted gene, whereas the second part is directed in sense. The insert is flanked by a 15 bp duplicated region. This insertion detected by long-range PCR escaped routine sequencing due to its size.

For patients P2, P3, and P4, breakpoints were narrowed down by primer walking. Patient P2 showed a decreased dosage for intron 11 and 14. Primers flanking the predicted deletion resulted in a PCR product spanning the deletion breakpoints. Sequence analysis revealed a 6.1 kb deletion between intron 11 and 14 (c.5374+9369_5950-254del6107bp). This deletion event occured within Alu elements. Figure 1b depicts a sequence alignment of the identified breakpoint junction in patient P2 and the wildtype 5' and 3' regions. The sequences are characterised by high degree of homology and the junction contains 28 bp of microhomology (boxed, Figure 1b).

In patient P3, altered gene dosages were observed within introns 10 and 15. Sequencing of PCR products spanning the deletion breakpoints revealed a 20.7 kb deletion (c.5274+13396_6107-3547del20729bp). The 5' breakpoint is located within a LINE1 element in intron 10 and the 3' breakpoint is placed between an AluY and a L1ME3 element in intron 15. Patient P4 was homozygous for this deletion. Sequences surrounding the deletion breakpoints in comparison to the junctions in patient P3 and patient P4 are depicted in Figure 1c. There was no evidence for sequence homology of the breakpoints.

Haplotype analyses of the complete 5.4 cM interval between D9S159 and D9S1793 did not show a common genotype for patient P3 and P4 (additional file 4). However, both patients shared a common allele for chromosome 9 markers D9S1847 and D9S1830 that are located next to the *SETX *gene.

### Expression of mutated *SETX *alleles

**Figure 2:**
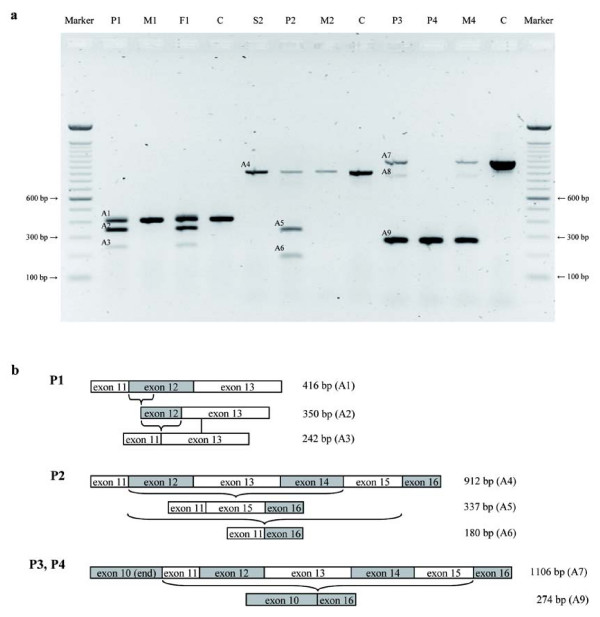
**Aberrant Transcripts identified by RT-PCR**. **(a) **RT-PCR products separated on a 0.8% agarose gel. Marker: 100 bp DNA Ladder, A1-A9: PCR products, M: mother, F: father, S: sister, C: control. **(b) **Schematic maps of the RT-PCR. Exons (boxes) are represented to scale.

In leukocytes of all four patients, aberrant transcripts could be identified by RT-PCR (Figure 2). Amplification of cDNA from exon 11 to exon 13 revealed two additional products (A2 and A3) in patient P1 and his father F1. The mother M1 and the control C carried the expected wildtype fragment (A1, Figure 2a). In addition to this wildtype fragment (416 bp, A1), sequence analysis yielded a transcript lacking the first 66 bp of exon 12 (350 bp, A2) and another transcript missing the complete exon 12 (242 bp, A3).

In patient P2, three fragments could be detected by RT-PCR using exonic primers from exons 11 and 16. In addition to the wildtype fragment A4 (912 bp) that was present in the cDNA of the healthy sister S2, the mother M2, and in the control sample, two shortened products lacking exons 12 to 14 (337 bp, A5) or exons 12 to 15 (180 bp, A6) could be found.

For patient P3, two products resulting from RT-PCR with primers amplifying exons 10 to 16 were subjected to further investigations: The upper 1106 bp fragment A7 represented the wildtype sequence. In the lower 274 bp fragment (A9), exon 10 was found to be spliced to exon 16.

Sequencing yielded an additional faint signal (A8) corresponding to a heteroduplex composed of A7 and A9. In the cDNA of patient P4, the 274 bp fragment (A9) was observed in the absence of the wildtype transcript. The mother M4 of patient P4 being heterozygous for the mutation, displayed the same expression pattern as patient P3, while the control sample exclusively showed the wildtype fragment.

## Discussion

In this study, we describe four patients with large mutations within the *SETX *gene. We have identified one insertion and two different deletions. Interestingly, all insertion and deletion events occurred within or near transposable elements (TE). Transposable elements are frequently recurring sequences spread all over the human genome. They comprise about 45% of the total genome sequence and can be categorized into four classes: short interspersed elements (SINE), long interspersed elements (LINE), LTR retrotransposons and DNA transposons [16].

The complex insertion in patient P1 showed homology to L1HS elements. L1HS elements are human specific LINE1 (L1) elements. Some full-length L1 elements are still capable of active retrotransposition. Retrotranspositionally competent L1 elements have reverse transcriptase and endonuclease activity [17]. L1-mediated insertions typically integrate at an endonuclease consensus cleavage site (3'-A/TTTT-5') and show characteristic hallmarks like 5' truncation, target site duplication and a long 3' poly A tail [18]. The 1.3 kb insertion detected in patient P1 has typical characteristics of L1-mediated retrotransposition. The retrotranspositional event occurred at the L1 endonuclease cleavage site 3'-A/TTTT-5'. The insert harbours a 5' truncation and a poly A tail. The entire element is flanked by a 15 bp target site duplication. L1 elements are thought to be inserted either in antisense or in sense orientation with respect to disrupted target genes [18]. However, in patient P1 the first part of the 5' truncated L1HS element was found to be orientated in antisense whereas the second part was directed in sense. This fact raises the question, if the insertion resulted from a single retrotranspositional event, or if the generation of this complex insertion is due to several independent steps. Irrespective of the generation of the insertion, this mutation seems to be stably inherited: The identical variation was also present in the father. Expression analyses revealed two aberrant splice variants for patient P1. Transcripts missing the first 66 bp of exon 12 as well as transcripts with loss of the complete exon 12 were detected. Both deletions are in-frame, therefore maintaining the open reading frame. The insertion seems to destroy the acceptor splice site of intron 11. In the case of the 66 bp deletion an alternative splice site within exon 12 is used.

The deletion breakpoints for the 6.1 kb deletion in patient P2 and the 20.7 kb deletion in patients P3 and P4 are located in or near transposable elements. The 6.1 kb deletion occurred between two Alu elements, whereas the 20.7 kb deletion involved LINE1 and Alu elements. Interestingly, the known ~10 kb duplication and the ~20.6 kb deletion [4,7] also occurred within or near Alu elements.

Alu elements represent the major subgroup of SINE elements. RT-PCR with primers flanking the 6.1 kb deletion showed two aberrant transcripts for patient P2. In one transcript exons 12 to 14 were deleted while exons 12 to 15 were missing in another transcript. The 6.1 kb deletion occurred between introns 11 and 14. The acceptor splice site of intron 14 still exists at genomic level and seems to be partly functional. RNA analyses of the 20.7 kb deletion in patients P3 and P4 revealed an additional transcript lacking exons 11 to 15. This result is consistent with the deletion spanning intron 10 to intron 15 at genomic level. Haplotype analyses may point to an ancient founder effect for the deletion in patient P3 and P4 originating from distinct parts of Germany due to the fact that the alleles for D9S1847 and D9S1830 are identical in both patients. Nevertheless, it should be underlined that these alleles are common in the German population (data not shown).

In all cases presented here, the gross mutations were found to be associated with transposable elements (TE). Interestingly, the ~10 kb duplication and the ~20.6 kb deletion reported before [4,7] occurred within or near Alu elements, too. Human genes bearing a TE content > 40% seem to have an increased frequency of gross deletions [16]. Furthermore, the deletion breakpoints are predominantly located in TE subclasses that are specifically overrepresented in the involved gene compared to the human genome [16]. Repetitive elements - mainly LINE1 and Alu elements - account for 47.5% of the entire *SETX *gene explaining the high number of deletions and insertions.

Only four of 51 *SETX *mutations identified so far have been reported to be gross mutations (7.8%) [4,7,14]. In our own series, three of 15 distinct *SETX *mutations (20%) were found to be large alterations. These discrepancies may result from methodological problems since gross mutations potentially escape routine diagnostics due to their size.

## Conclusion

Gross mutations potentially may escape routine diagnostic due to their size. Thus, large deletions, insertions, and duplications are probably an underestimated cause for AOA2.

## Competing interests

The authors declare that they have no competing interests.

## Authors' contributions

CZ designed the study. VB carried out the molecular genetic work. MM, KB and FK investigated the patients and collected the clinical data. GG participated in the design of the study and helped to draft the manuscript. All authors read and approved the final manuscript.

## Pre-publication history

The pre-publication history for this paper can be accessed here:



## Supplementary Material

Additional file 1 **Primer sequences for chromosome 9 markers**. This file contains the primer sequences used for haplotype analyses. Click here for file

Additional file 2 **Primer sequences for breakpoint localisation**. This file contains the primer sequences used for breakpoint localisation. Click here for file

Additional file 3 **Primer sequences for RT-PCR**. This file contains the primer sequences used for RT-PCR. Click here for file

Additional file 4 **Linkage analysis for patient P3 and patient P4**. This file shows the linkage analysis for patient P3 and patient P4. Chromosome 9 haplotypes between markers D9S159 and D9S1793 are shown. Click here for file
