# Supplementary material for: Exon deletions and intragenic insertions are not rare in ataxia with oculomotor apraxia 2
Source: BMC Med Genet. 2009 Sep 11;10:87. doi: 10.1186/1471-2350-10-87 (PMC2749023; doi:10.1186/1471-2350-10-87)
Supplement: Additional file 1 — Primer sequences for chromosome 9 markers. This file contains the primer sequences used for haplotype analyses. [file 1471-2350-10-87-S1.doc]

| Marker | Primer sequences |
| --- | --- |
| D9S159 | 5’- cacgacgttgtaaaacgacctttctgacggcagccaggt -3’  5’- agctggaatgagtgctgggc -3’ |
| D9S1831 | 5’- cacgacgttgtaaaacgaccagtgaatcgaggtcgc -3’  5’- ccagtgttaaagtcagccg -3’ |
| D9S1863 | 5’- cacgacgttgtaaaacgacttgggccacccataaaata -3’  5’- ctagagacccaggcaattctt -3’ |
| D9S1847 | 5’- cacgacgttgtaaaacgactacagcgccagatttgg -3’  5’- ggcaggagccgtgtgt-3’ |
| D9S1830 | 5’- cacgacgttgtaaaacgaccagagtggtgggactcaa -3’  5’- agctgcagactgccttc-3’ |
| D9S1793 | 5’- cacgacgttgtaaaacgacggtgtggaaccaggactaac-3’  5’- cagagcgagtgtaatccg-3’ |
