# Supplementary material for: Exon deletions and intragenic insertions are not rare in ataxia with oculomotor apraxia 2
Source: BMC Med Genet. 2009 Sep 11;10:87. doi: 10.1186/1471-2350-10-87 (PMC2749023; doi:10.1186/1471-2350-10-87)
Supplement: Additional file 2 — Primer sequences for breakpoint localisation. This file contains the primer sequences used for breakpoint localisation. [file 1471-2350-10-87-S2.doc]

| Mutation | Primer sequence | Position | Product [bp] |
| --- | --- | --- | --- |
| c.5401_5402ins1280bp | 5’- gcagagtcctttagtgctgtg -3’ | intron 11 | 1634  (wildtype 354) |
|  | 5’- gattattagatgagactgtatctg -3’ | intron 12 |  |
| c.5374+9369_5950-254del6107bp | 5’- gagtgcaatggcgcaatcttg -3’ | intron 11 | 359 |
|  | 5’- gcatctacatctaacactg -3’ | intron 14 |  |
| c.5274+13396_6107-3547del20729bp | 5’- gtatagccatcaccacaacccag-3’ | intron 10 | 586 |
|  | 5’- gagcttgctaatgcccacaag -3’ | intron 15 |  |
