# Supplementary material for: Exon deletions and intragenic insertions are not rare in ataxia with oculomotor apraxia 2
Source: BMC Med Genet. 2009 Sep 11;10:87. doi: 10.1186/1471-2350-10-87 (PMC2749023; doi:10.1186/1471-2350-10-87)
Supplement: Additional file 3 — Primer sequences for RT-PCR. This file contains the primer sequences used for RT-PCR. [file 1471-2350-10-87-S3.doc]

| Mutation | Primer sequence |
| --- | --- |
| c.5401_5402ins1280bp | 5’- cacaagaatggctcaactctc -3’ |
|  | 5’- actacccaacagagacatggc -3’ |
| c.5374+9369_5950-254del6107bp | 5’- cacaagaatggctcaactctc -3’ |
|  | 5’- ggctgtccaaactgaactttag -3’ |
| c.5274+13396_6107-3547del20729bp | 5’- gtctgtctctgacaccttcg -3’ |
|  | 5’- ggctgtccaaactgaactttag -3’ |
