# Supplementary figures and images for: Exon deletions and intragenic insertions are not rare in ataxia with oculomotor apraxia 2
Source: BMC Med Genet. 2009 Sep 11;10:87. doi: 10.1186/1471-2350-10-87 (PMC2749023; doi:10.1186/1471-2350-10-87)

## Slide 1
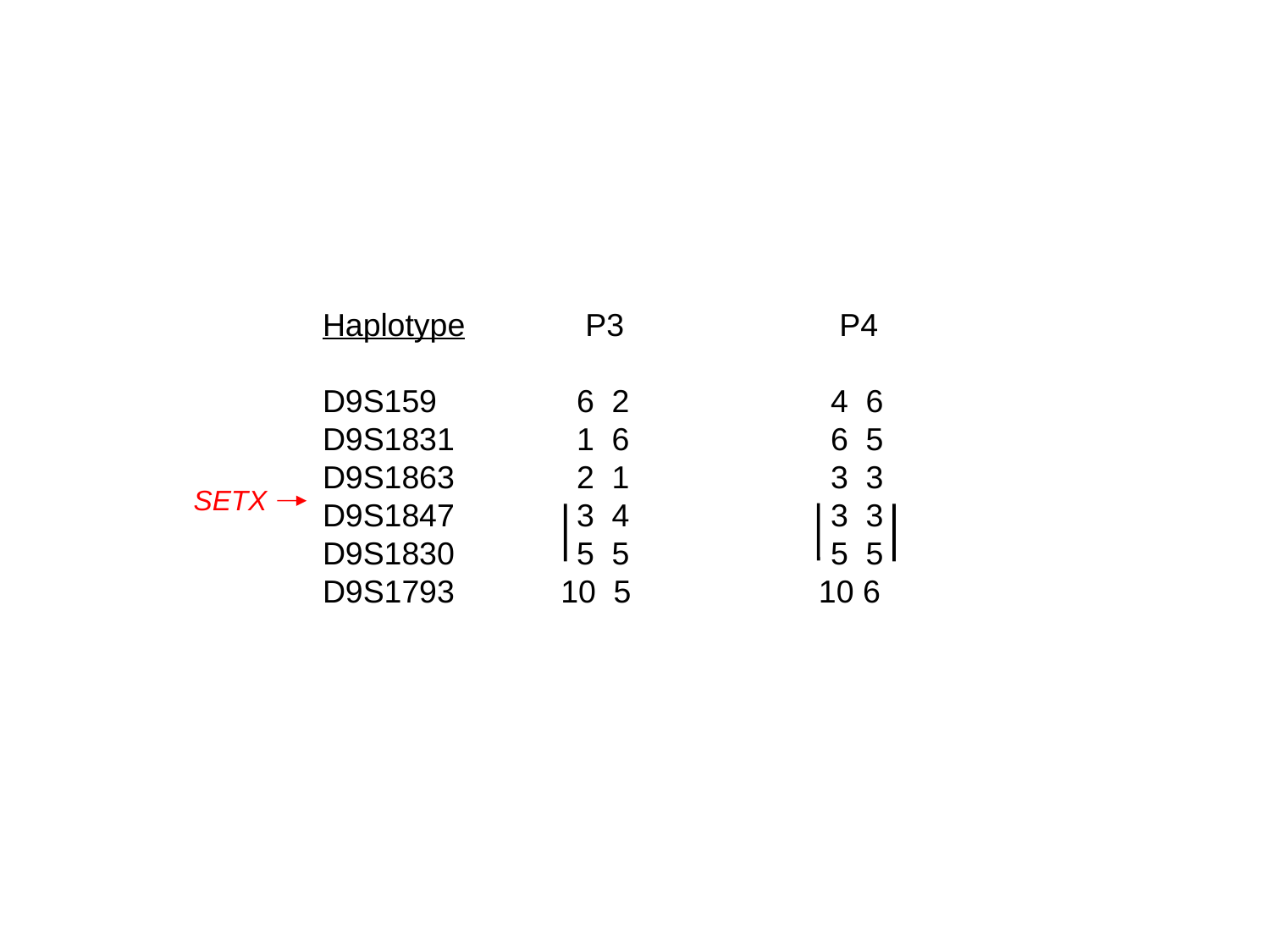

Haplotype	 P3		 P4
D9S159		6 2		4 6
D9S1831	1 6		6 5
D9S1863	2 1		3 3
D9S1847	3 4		3 3
D9S1830	5 5		5 5
D9S1793 10 5	 10 6
SETX

Supplement: Additional file 4 — Linkage analysis for patient P3 and patient P4. This file shows the linkage analysis for patient P3 and patient P4. Chromosome 9 haplotypes between markers D9S159 and D9S1793 are shown. [file 1471-2350-10-87-S4.ppt]
